# Predicting Specificities Under the Non-self Gametophytic Self-Incompatibility Recognition Model

**DOI:** 10.3389/fpls.2019.00879

**Published:** 2019-07-04

**Authors:** Jorge Vieira, Sara Rocha, Noé Vázquez, Hugo López-Fernández, Florentino Fdez-Riverola, Miguel Reboiro-Jato, Cristina P. Vieira

**Affiliations:** ^1^Instituto de Biologia Molecular e Celular (IBMC), Universidade do Porto, Porto, Portugal; ^2^Instituto de Investigação e Inovação em Saúde, Universidade do Porto, Porto, Portugal; ^3^Escuela Superior de Ingeniería Informática (ESEI), Edificio Politécnico, Universidad de Vigo, Ourense, Spain; ^4^Centro de Investigaciones Biomédicas (Centro Singular de Investigación de Galicia), Vigo, Spain; ^5^SING Research Group, Instituto de Investigación Sanitaria Galicia Sur (IIS Galicia Sur), SERGAS-UVIGO, Vigo, Spain

**Keywords:** Solanaceae, SLFs, S-RNase, self-incompatibility, specificity recognition, positive selection, BDBM

## Abstract

Non-self gametophytic self-incompatibility (GSI) recognition system is characterized by the presence of multiple F-box genes tandemly located in the *S*-locus, that regulate pollen specificity. This reproductive barrier is present in Solanaceae, Plantaginacea and Maleae (Rosaceae), but only in *Petunia* functional assays have been performed to get insight on how this recognition mechanism works. In this system, each of the encoded *S*-pollen proteins (called SLFs in Solanaceae and Plantaginaceae /SFBBs in Maleae) recognizes and interacts with a sub-set of non-self *S*-pistil proteins, called S-RNases, mediating their ubiquitination and degradation. In *Petunia* there are 17 *SLF* genes per *S*-haplotype, making impossible to determine experimentally each SLF specificity. Moreover, domain –swapping experiments are unlikely to be performed in large scale to determine *S*-pollen and *S*-pistil specificities. Phylogenetic analyses of the Petunia SLFs and those from two *Solanum* genomes, suggest that diversification of *SLF*s predate the two genera separation. Here we first identify putative *SLF* genes from nine *Solanum* and 10 *Nicotiana* genomes to determine how many gene lineages are present in the three genera, and the rate of origin of new *SLF* gene lineages. The use of multiple genomes per genera precludes the effect of incompleteness of the genome at the *S*-locus. The similar number of gene lineages in the three genera implies a comparable effective population size for these species, and number of specificities. The rate of origin of new specificities is one per 10 million years. Moreover, here we determine the amino acids positions under positive selection, those involved in SLF specificity recognition, using 10 *Petunia S*-haplotypes with more than 11 *SLF* genes. These 16 amino acid positions account for the differences of self-incompatible (SI) behavior described in the literature. When SLF and S-RNase proteins are divided according to the SI behavior, and the positively selected amino acids classified according to hydrophobicity, charge, polarity and size, we identified fixed differences between SI groups. According to the *in silico* 3D structure of the two proteins these amino acid positions interact. Therefore, this methodology can be used to infer SLF/S-RNase specificity recognition.

## Introduction

To avoid self-fertilization and promote out-crossing, many Angiosperms have developed self-incompatible (SI) mechanisms, that allow the pistil to reject self-pollen and thus, only non-genetically related pollen is allowed to effect fertilization (De Nettancourt, 1977). Despite the large diversity of SI mechanisms, the most common pre-zygotic reproductive system in flowering plants is the gametophytic self-incompatibility (GSI) system (Igic et al., 2008). The genes determining pistil (*S*-pistil) and pollen (*S*-pollen) GSI specificity have been characterized in self-incompatible Papaveraceae, Solanaceae, Plantaginaceae, and Rosaceae species. In Papaveraceae, the pollen-pistil interaction occurs in the stigma surface during or shortly after germination (Foote et al., 1994). The pistil *S*-locus is a small protein (∼15 kDa, called PrsS (*Papaver rhoeas* stigmatic *S*- protein; Foote et al., 1994) secreted to the pistil surface that binds to the self- pollen *S*-receptor [called PrpS (*Papaver rhoeas* pollen *S*); Wheeler et al., 2009], triggering a Ca (2+) dependent signaling network, that results in pollen inhibition and programmed cell death (Eaves et al., 2014; Wilkins et al., 2014). In Solanaceae, Plantaginaceae, and Rosaceae the rejection of self-pollen occurs during the growth of pollen tubes in the style, and the pistil gene is an extracelular ribonuclease, called *S-RNase* (Roalson and McCubbin, 2003; McClure, 2009; Nowak et al., 2011), and the pollen gene(s) is(are) F-box protein(s) (Entani et al., 2003; Ushijima et al., 2003; Qiao et al., 2004; Tsukamoto et al., 2005, 2010; Cheng et al., 2006; Nunes et al., 2006; Hua et al., 2007; Sassa et al., 2007; Wheeler and Newbigin, 2007; Kubo et al., 2010, 2015; Minamikawa et al., 2010; Kakui et al., 2011; Okada et al., 2011; Aguiar et al., 2013, 2015; Williams et al., 2014). Therefore, Papaveraceae GSI has evolved independently from that of Solanaceae, Plantaginaceae, and Rosaceae. In Solanaceae, Plantaginaceae, and Rosaceae, both phylogenetic analyses and gene structure analyses (conserved and hypervariable regions, intron number and position) of the *S-RNase*, shows that the RNase-based system has evolved only once, about 120 million years ago, before the separation of the Asteridae and Rosideae (Igic and Kohn, 2001; Steinbachs and Holsinger, 2002; Vieira et al., 2008; Ramanauskas and Igic, 2017). The shared ancestry, however, does not imply that gene duplications, followed by functional change, could occur and thus, that paralogous genes could be determining *S*-pistil specificity in different species groups. Indeed, in the Rosaceae family, two gene lineages are determining *Prunus* (Amygdaleae) and *Malus* (Maleae) *S*-pistil and *S*-pollen specificity (Aguiar et al., 2015). In these species a self- and non-self- recognition mechanisms is present, respectively. So far, only *Prunus* has an *RNase* based self-recognition mechanism. In this system, only one *S*-pollen gene (called *SFB*), is sufficient to account for the self-S-RNase inhibition (Entani et al., 2003; Ushijima et al., 2003; Ikeda et al., 2004; Tsukamoto et al., 2005, 2010; Nunes et al., 2006; Hua et al., 2007; Aguiar et al., 2015). In this model, a “general inhibitor,” such as the gene products of the F-box like genes in the vicinity of the *S*-locus region (Matsumoto and Tao, 2016; Chen et al., 2018), inactivate the cytotoxic effect of non-self S-RNases (Luu et al., 2001; Sonneveld et al., 2005).

In the non-self -recognition mechanism multiple *S*-pollen genes (called *SLFs*; *S*-locus F-box in Solanaceae (Wheeler and Newbigin, 2007; Kubo et al., 2010, 2015; Williams et al., 2014, 2015; Sun et al., 2015; Li et al., 2017; Wu et al., 2018), and *SFBB*; *S*-locus F-box brothers in Maleae (Sassa et al., 2007; Minamikawa et al., 2010; Kakui et al., 2011; Okada et al., 2011; Aguiar et al., 2013, 2015)) regulate pollen specificity. Each of the encoded *S*-proteins recognizes and interacts with a sub-set of non-self S-RNases, and mediate their degradation, under a model called the collaborative non-self -recognition model (Kubo et al., 2010, 2015; Aguiar et al., 2013; Williams et al., 2014, 2015; Sun et al., 2015; Pratas et al., 2018).

*Petunia inflata* and *P. axillaris* (Solanaceae) are the model species for functional assays on how the non-self -recognition mechanism works. Transgenic *P. inflata* has been used to show that S-RNases most likely function as a toxin that specifically degrades RNA of incompatible pollen tubes. Indeed, the expression of a mutant form of S3-RNase (where the His residue in the catalytic domain has been replaced with Arg), in a plant with the *S1S2* genotype did not confer the ability of the pistils of this transgenic plant to reject *S3* pollen (Huang et al., 1994). Nevertheless, transgenic plants carrying pollen from two different *S*-haplotypes are self-compatible. This “competitive interaction” gain of function observation was used to identify the first *Petunia SLF* gene (Sijacic et al., 2004). Transgenic assays using the first identified *SLF* gene in different *S*-haplotype backgrounds did not produce self-compatible plants, leading to the identification of other *SLF* genes involved in GSI specificity (Kubo et al., 2010).

The characterization of the behavior of the products of three *SLF* genes, in the context of four different *S*-haplotypes revealed that only a sub-set of SLFs interact with a given non-self S-RNase (Kubo et al., 2010). This implies a large number of *SLF* genes to account for the large number of *S-RNases* [about 32 in *P. inflata* and *P. hybrida*, that are genetically distinct; (Sims and Robbins, 2009)]. Other approaches have been used, such as the use of artificial microRNA to determine which S-RNases are recognized by the product of a particular *SLF* allele (Sun and Kao, 2013). Co-immunoprecipitation experiments also show that non-self *S*-pollen proteins and the S-RNases interact during the SI response (Kubo et al., 2010). These experiments show that SLFs are in an SCF complex, as predicted by the protein degradation model in which SLFs are the F-box proteins of the E3 ubiquitin ligase complex that mediates ubiquitination of all non-self S-RNases (Hua and Kao, 2006; Hua et al., 2008).

To get insight into how many SLFs can contribute for the *S*-pollen specificity determination, Williams et al. (2014) have analyzed the pollen transcriptome of two *S*-locus homozygous plants. These authors found 17 *SLF* genes for each *S*-haplotype, seven of which were already known (Kubo et al., 2010; Williams et al., 2014). For 11 of these genes, segregation analyses support their role in *S*-pollen specificity determination (Kubo et al., 2010; Williams et al., 2014). Using large-scale next-generation sequencing and genomic PCR techniques, the characterization of *SLF* genes in eight *P. hybrida S*-haplotypes revealed that the number of *SLF* genes per *S*-haplotype varies from 16 to 20, that have been grouped into 18 types (Kubo et al., 2015). Thus, different related genes are grouped into the same *SLF* type. The apparent variation in copy number suggests that SLF specificity recognition can either be achieved by a diverged or deleted allele of a *SLF* gene (Kubo et al., 2015). Indeed, using this observation alone, these authors could predict the SLF specificity of an allele for seven S-RNases.

Phylogenetic analyses based on the annotation of the *Solanum lycopersicum* and *S. tuberosum* genomes as well as custom annotations, led to the identification of 13 and 14 genes, respectively. These analyses suggested that diversification of *SLF*s predated the separation of the *Petunia* and *Solanum* genera, although a small number of *SLF*s are genera specific (Kubo et al., 2015). Since the number of specificities that can be maintained in a population depends on the effective population size of a population/species (Wright, 1939), the difference found could just reflect different effective population sizes of the genera analyzed. Reliable estimates of the rate at which *SLF* genes emerge is fundamental to understand how the system evolves. This can only be done using a large number of species from different genera. Thus, in this work we used nine genome assemblies from eight *Solanum* species and 10 genomes from seven *Nicotiana* species publicly available. Only four of these are self-incompatible species, and thus we searched for both genes and pseudogenes, using Blast DataBase Manager (BDBM^1^; Vázquez et al., 2019). The high number of genomes analyzed also implies that it is unlikely that a given *SLF* will be missing in all genomes simply by chance.

Identification of the regions involved in GSI specificity is also fundamental to understand how the system works. Domain-swapping transgenic experiments with similar *S-RNase* alleles have showed that the two hypervariable regions of *S-RNases*, are involved in specificity determination (Ide et al., 1991; Matton et al., 1997, 1999; Matsuura et al., 2001). Although there are positively selected amino acid sites in these regions, there are other amino acid sites in other regions of the *S*-pistil gene, that may be involved in recognition of the *S*-pollen gene(s) (Vieira et al., 2007; Brisolara-Correa et al., 2015). To address *S*-pollen specificity, *Petunia SLF1* gene has been divided into three functional domains (FD1 from amino acid position 1 to 110; FD2, from amino acid position 110 to 260, and FD3 from amino acid position 260 to 389), and *in vivo* assays performed. These experiments showed that FD2 is primarily responsible for the strong interaction between an allelic product of *SLF1* gene and the *S-RNase*, and this interaction is negatively modulated by FD1 and FD3 that together determine the specificity of the *SLF1* allele (Hua et al., 2007; Fields et al., 2010). Moreover, yeast two hybrid assays show that the C-terminal regions of SLF interact with S-RNases mainly in hypervariable regions, as also obtained in the 3D structural model of interaction of the SLFs and of S-RNase (Li et al., 2017). In the latter study Li and co-authors show, using *in vivo* domain-swapping transgenic experiments, that the SLF1 C-terminal region (amino acids 265–389) acts as a major specificity domain *in vivo*. Two amino acid sites (293 and 317) in this region show evidence for positive selection, using 21 sequences from *P. inflata*, *P. hybrida*, and *P. axillares* using PAML (Li et al., 2017), despite the prediction that, under the non-self-recognition model, natural selection favors diversification of *SLF* genes rather than alleles within an S haplotype (Kakui et al., 2011; Aguiar et al., 2013). Such analyses are here performed for 10 *Petunia S*-haplotyes for which more than 11 *SLF* genes are available, and the results deposited at the B+ database^2^ (Vázquez et al., 2017, 2018). The 16 amino acid positions under positive selection here identified account for the differences described in SI behavior when different alleles of the same *SLF* gene are analyzed (Kubo et al., 2010, 2015; Williams et al., 2014; Li et al., 2017). Indeed, we classify SLF specificity according to these amino acids sites. We characterized the S-RNase and SLF amino acid properties (hydrophobicity, charge, polarity and size) at positions under positive selection, to infer critical features for GSI specificity determination. The identification of these amino acid sites on the three-dimensional structural model of interaction of the *S*-pistil and *S*-pollen proteins shows that the two proteins interact at these amino acid sites.

## Materials and Methods

### Solanaceae *SLF*s Dataset

*Petunia SLF*s were obtained by querying the NCBI nucleotide database^3^ with the words “Petunia” and “SLF.” We complemented this approach by looking for those *Petunia SLF* genes listed in Supplementary Table 1 of Kubo et al. (2015) and Williams et al. (2014), that were not retrieved because the word SLF is missing in the NCBI record. The acc. numbers of the 244 CDS used are shown in brackets in Supplementary Figure S1.

To select reference sequences to be used for the identification of *SLF* CDS in Solanaceae species, we obtained a Bayesian phylogenetic tree [using MrBayes; (Huelsenbeck and Ronquist, 2001)], after using Muscle as implemented in T-coffee (Notredame et al., 2000), to align the sequences, as implemented in ADOPS (Reboiro-Jato et al., 2012). The GTR model of sequence evolution that was used, allowed for among-site rate variation and a proportion of invariable sites. Third codon positions were allowed to have a gamma distribution shape parameter different from that of first and second codon positions. Two independent runs of 1,000,000 generations with four chains each (one cold and three heated chains) were set up. Trees were sampled every 100th generation and the first 2500 samples were discarded (burn-in). The remaining trees were used to compute the Bayesian posterior probabilities of each clade of the consensus tree. The potential scale reduction factor for every parameter was about 1.00 showing that convergence has been achieved. The tree was rooted with a *SLF-like* gene (a gene (*S2-SLF-like1*) not involved in GSI specificity determination from *P. integrifolia*). For each of the identified genes we selected a reference sequence (marked in bold in Supplementary Figure S1).

Nine genomes from eight *Solanum* species and 10 genomes from seven *Nicotiana* species (Table 1) were downloaded from NCBI (assembly database). For those genomes having an annotation, the corresponding predicted CDSs were also downloaded (Table 1). Using the Blast DataBase Manager software (BDBM; Vázquez et al., 2019) *Reformat FASTA* option, sequence headers were reformatted in order to show the species name and accession numbers only. Since *SLF* genes are always intronless (Wang et al., 2004), then we obtained all open reading frames (ORFs) from these genomes using the *Get ORF* operation of BDBM (that uses the getorf command of EMBOSS; Vázquez et al., 2019), with a minimum and maximum size of 300 and 10000 bp, respectively. We then prepared a Blast database for each of the resulting FASTA files, using the *Make Blast Database* option of BDBM (Vázquez et al., 2019). Moreover, using the same software, we created an alias in order to treat all databases of interest as a single one (using *BLAST DB Alias* operation as implemented in BDBM; Vázquez et al., 2019). A tblastn search was then performed, using as the query the 36 translated *Petunia SLF* reference sequences without the F- box motif (the first 60 amino acids), and an e-value of 0.05. This approach will retrieve nucleotide sequences showing similarity at the amino acid level with the SLF reference sequences beyond the F-box motif. To the obtained dataset we added the predicted CDSs from the annotated genomes, and removed identical sequences, after merging the headers, using SEDA^4^ (López-Fernández et al., 2019). As before, sequences were aligned using Muscle and a Bayesian phylogenetic tree was obtained using ADOPS (Reboiro-Jato et al., 2012).

**Table 1:** Breeding system for the species used in this work.

Species	Breeding system	References
*Solanum commersonii*	SI	Igic et al., 2006
*S. melongena*	SC	Igic et al., 2006
*S. arcanum*	SC and SI	Igic et al., 2008
*S. habrochaites*	SC and SI	Igic et al., 2006; Broz et al., 2017
*S. pimpinellifolium*	SC	Igic et al., 2006
*S. tuberosum*	SC	Igic et al., 2006
*S. pennellii*	SC and SI	Igic et al., 2006
*S. lycopersicum*	SC	Igic et al., 2006
*Nicotiana attenuata* (2)	SC	Igic et al., 2006
*N. benthamiana*	SC	Goldberg et al., 2010
*N. obtusifolia*	SC	Goldberg et al., 2010
*N. otophora*	SC	Igic et al., 2006
*N. sylvestris*	SC	Igic et al., 2006
*N. tabacum* (3)	SC	Moon et al., 2008
*N. tomentosiformis*	SC	Igic et al., 2006

*S. arcanum is strictly SI, apart from one known SC accession LA2157, here used, which expresses an inactive S-RNase, due to the loss of the histidine residue at the active site of the enzyme (Kowyama et al., 1994; Royo et al., 1994); S. pennellii LA0716, here used, is also SC (Liedl et al., 1996) and does not express the S-RNase protein (Chalivendra et al., 2013; Li and Chetelat, 2014). S. habrochaites LYC4, here used, is also SC (Markova et al., 2016). In brackets are the number of genomes used.*

*SLF* genes may be missed when using the above mentioned approach, if there are mistakes in the genome sequence, such as insertions and deletions. Moreover, most species here studied are self-compatible (Table 1), and thus the coding region of many SLFs may not be a multiple of three, meaning that they are pseudogenes. Since one of the objectives of this research is to identify all *SLF* gene lineages present in the Solanaceae genomes, we also used the *Splign-Compart* and the *ProSplign-Compart* options (that are insensitive to frameshifts since the annotation is based on similarity at the nucleotide or amino acid level, respectively, and presence of putative splice sites only), as implemented in BDBM (Vázquez et al., 2019). As reference sequences we used for Splign-Compart all sequences shown in Supplementary Figure S2, and for ProSplign-Compart the translation of these sequences. In order to address the hypothesis that there are Solanaceae *SLF* gene lineages that are not present in *Petunia*, the *P. inflata* and *P. axillaris* genomes were also downloaded from Sol Genomics Network^5^, and the Splign-Compart as well as the ProSplign-Compart options (implemented in BDBM; Vázquez et al., 2019) used.

### Phylogenetic and Positive Selection Analyses

Sequences were aligned using Muscle and a Bayesian phylogenetic tree obtained using MrBayes (Huelsenbeck and Ronquist, 2001), as described above.

Positively selected amino acid sites were inferred using codeML (Yang, 2007) as implemented in ADOPS (Reboiro-Jato et al., 2012), using 10 *Petunia S*-haplotypes for which, according to (Williams et al., 2014; Kubo et al., 2015), more than 11 *SLF* genes are available. Phylogenetic trees were obtained as described above. All details can be seen at the B+ database (bpositive.i3s.up.pt; Petunia dataset BP2017000006). Models comparisons were M2a-M1a and M8-M7. We consider as positively selected those amino acid sites that show a probability higher than 95% for both naive empirical Bayes (NEB) or Bayes empirical Bayes (BEB) methods in at least one of the analyses.

### Protein Prediction Analyses

The protein sequence of *P. integrifolia* S3-RNase (M67991), *P. hybrida* S7-RNase (AB568388), *P. axillaris* S19-RNase (Kubo et al., 2010), *P. integrifolia* S2-SFL1 (AY500391), *P. hybrida* S5-SFL3 (AB568399), and *P. hybrida* S11-SFL9 (AB933017) were obtained from NCBI. In the N-terminal region of S3-RNase, S7-RNase, and S19-RNase, we removed the first 23 amino acid residues, corresponding to the peptide cleavage site, according to SignalP 4.1 server (Nielsen, 2017). In the N-terminal region of the S5-SLF1, S7-SLF1, S9-SLF1 and S17-SLF1 we removed the first 49 amino acid residues representing the F-box domain. The 3D structure predictions for all proteins (S-RNases without signal peptide and S-SLFs without F– box domains) were modeled by I-Tasser (Yang et al., 2015). For all proteins we used the models with the highest C-score values. The docking of the two proteins was inferred using the HADDOCK server (van Zundert et al., 2016). For both the S-RNase and SLFs, the active residues used in HADDOCK were the amino acids under positive selection (for the S-RNase see (Vieira et al., 2007); for the SLFs see Results) and the two surrounding amino acids. In the case of SLFs we also used the amino acids described in Li et al. (2017) and Wu et al. (2018) as being involved in specificity determination. The passive residues for S-RNases and SLFs were automatically defined around the active residues.

For all HADDOCK clusters showing negative Z-Scores, PISA (Krissinel and Henrick, 2007) was used to calculate the number of hydrogen bonds and salt bridges for the *P. hybrida* S19-RNase:S11-SFL9. The cluster with the highest number of hydrogen bonds and salt bridges was chosen as being the most likely. This result was used as reference for the inferences regarding the docking of the other S-RNases and SLFs, such as *P. integrifolia* S3-RNase:S2-SFL1, and *P. hybrida* S7-RNase:S5-SFL3. This was achieved by selecting from the HADDOCK clusters with negative Z-Scores the one that presented the best structural similarity (according to TM-score) with the chosen model structure of *P. hybrida* S19-RNase:S11-SFL9, using the TM-align server (Zhang and Skolnick, 2005). All structural images were produced using PyMOL (The PyMOL Molecular Graphics System, Version 1.7.4 Schrödinger, LLC).

## Results

### *SLF* Gene Number in Three Solanaceae Lineages (*Petunia*, *Solanum*, and *Nicotiana*)

**FIGURE 1:**
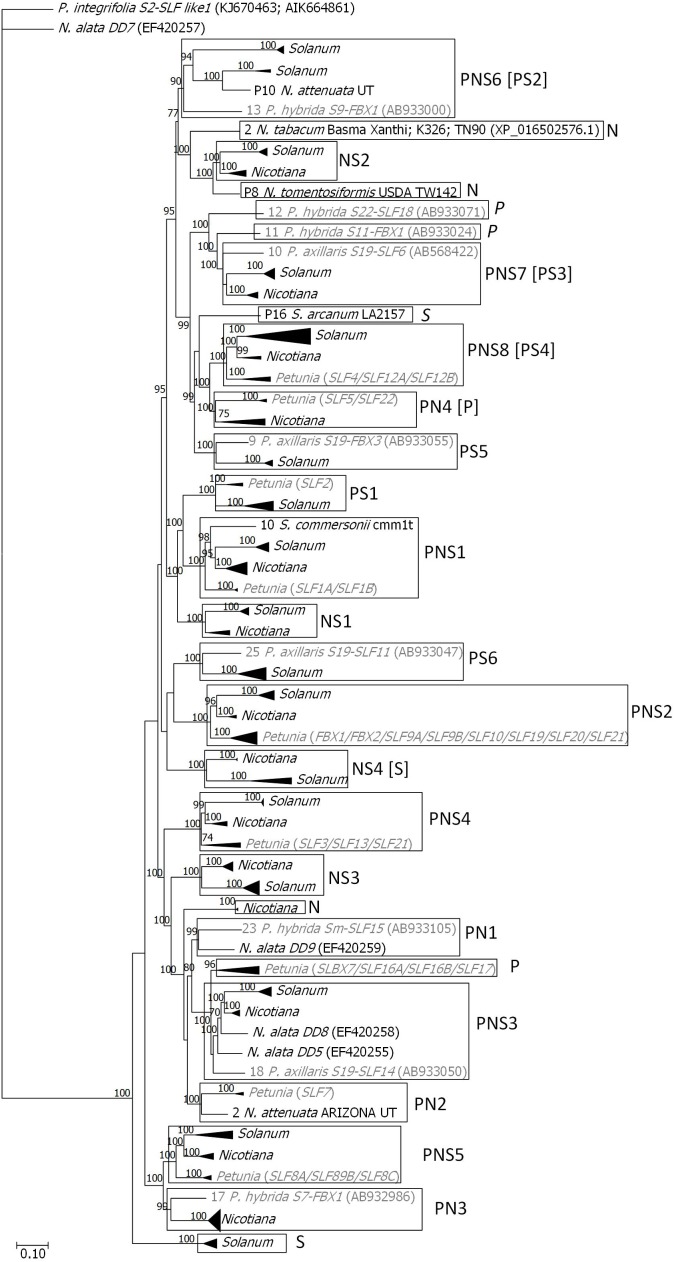
Bayesian phylogenetic tree showing the relationship of the *Petunia*, *Solanum*, and *Nicotiana SLF* sequences, used to infer gene lineages. The tree was rooted with *P. integrifolia S2-SLFlike1* (KJ670463; AIK66486). For *Petunia* (in gray) only one sequence for each *SLF* gene (1–36; Supplementary Figure S1) was used. Squares represent gene lineages, and P, N, and S are used to identify if the gene lineage is present in *Petunia*, *Nicotiana*, and *Solanum*, respectively. In square brackets is the characterization of each gene lineage when pseudogenes are not considered (Supplementary Figure S2). Numbers above the branches represent posterior credibility values above 70.

In order to estimate how many of the lineages present in *Petunia* predate the separation of the different Solanaceae species, we need to establish first a set of *Petunia* nucleotide sequences representative of each gene lineage. The phylogenetic relationship of the 245 *SLF Petunia* sequences imply a minimum of 36 *Petunia SLF* genes (Supplementary Figure S1). Of these genes, nine (2, 6, 8, 14, 20, 29, 31, 32, 33 in Supplementary Figure S1) are recent duplications and may be restricted to *Petunia* species. Five of these genes (9, 11, 13, 17, and 22 in Supplementary Figure S1) represent old lineages and thus were expected to be present in most haplotypes, but have been reported for one *S*-haplotype only. The Bayesian phylogenetic tree obtained using the 29 *Nicotiana* and 75 *Solanum SLF* sequences obtained after using the *Get ORF* option of BDBM (Vázquez et al., 2019; using the nine available genomes for eight *Solanum* species, and the 10 available genomes for seven *Nicotiana* species; Table 1; see Material and Methods), together with one sequence of each *Petunia* gene, and the nine *N. alata SLF* sequences (named *DD*s; Wheeler and Newbigin, 2007), revealed five gene lineages common to the three genera (marked as PNS in Supplementary Figure S2), six present in *Petunia* and *Solanum* (PS in Supplementary Figure S2), three in *Petunia* and *Nicotiana* (PN in Supplementary Figure S2), and three in *Nicotiana* and *Solanum* (NS in Supplementary Figure S2). Therefore, most of the gene lineages seem to be missing at one of the genera. Nevertheless, since most species analyzed are SC, *SLF*s may be pseudogenes in these species and have frameshifts that precluded their identification using the *Get ORF* option. Indeed, when using the *Splign-Compart* option, as implemented in BDBM (Vázquez et al., 2019), 59 *SLF* sequences are identified (marked in bold in Supplementary Figure S3). The number of gene lineages present in the three genera is then eight (marked as PNS; Figure 1), common to *Petunia* and *Solanum* there are three, in *Petunia* and *Nicotiana* there are four, and in *Nicotiana* and *Solanum* there are four. There are three divergent lineages present in *Nicotiana*, and two in *Solanum*, as observed in *Petunia*. Therefore, the number of *SLF* genes (17 to 19) is similar in the three genera. This implies that most of the *Petunia* gene duplications have occurred after the genus split, and are *S*-haplotype specific. The two *Petunia* genomes here analyzed revealed only three known *SLF* genes (*SLF2*, *SLF7*, and *SLF17*; Figure 1). The sequence identified in *P. axillaris* genome is likely from a novel haplotype, since there are 49 differences between the sequence we retrieved and the *P. axillaris S19*-*SLF2* (AB933033) sequence. For *P. inflata* the *SLF17* sequence is 100% identical to *P. integrifolia S2-SLF17* (KJ670458), but the *SLF7* sequence is only 99% identical (there are eight nucleotide differences between the two sequences) to the *P. integrifolia S2-SLFla* (EF614189; an allele of *SLF7*; Supplementary Figure S1). It is conceivable that the sequence we retrieved comes from a yet to be characterized *S*-haplotype, but it is also possible that the observed differences are due to sequencing or assembly mistakes. Nevertheless, this approach shows that the *Petunia* genomes here used either have a low coverage or the *S*-locus region is difficult to assemble in *Petunia*.

For six genomes, including three from SI or SI/SC *Solanum* species, the number of sequences identified is 16 or more (Table 2), a number similar to that observed in *Petunia* (Kubo et al., 2015). Only for the SI species *S. habrochaites* the number of identified *SLF* sequences is 13. For the SI species, a maximum of three sequences were inferred to be non-functional. The *Solanum* sequences that belong to the PNS6 and PNS8 lineage (Supplementary Figure S3) are mostly non-functional and thus are unlikely to be inferred to be pseudogenes because of sequencing errors. In the other cases, however, this is a possibility. In *N. tabacum* at least 20 *SLF* sequences have been identified, but half are non-functional, which is expected since this is a SC species. In conclusion, the number of *SLF* genes per *S*-haplotype in *Solanum* and *Nicotiana* seem to be similar to that of *Petunia*, implying similar effective population sizes for the three species.

**Table 2:** Number of *SLF* sequences (including pseudogenes) and size for the genomes analyzed.

Species	N	Non-functional	Size (bp)	
			Minimum	Maximum
*S. commersonii*	16	3	909	1295
*S. melongena*	2	0	1169	1221
*S. arcanum*	17	3	1131	1311
*S. habrochaites*	13	1	1140	1308
*S. pimpinellifolium*	10	3	1062	1311
*S. tuberosum*	15	1	1140	1281
*S. pennellii*	16	3	1131	1311
*S. lycopersicum* Heinz	12	9	1062	1266
*S. lycopersicum* M82	13	9	990	1266
*N. attenuata* UT	11	7	936	1199
*N. attenuata* Arizona	5	2	936	1181
*N. benthamiana*	0		0	0
*N. obtusifolia*	1		1161	-
*N. otophora*	7	1	1140	1293
*N. sylvestris*	11	9	1028	1299
*N. tabacum* Basma	20	10	1028	1299
*N. tabacum* k326	21	12	1028	1299
*N. tabacum* TN90	21	11	1028	1299
*N. tomentosiformis*	8	5	1140	1272

*N is the total number of sequences.*

### On the Identification of the Amino Acid Sites Determining *S*-Pollen GSI Specificity

Positively selected amino acid sites have been used to infer the amino acid positions that are, in principle, responsible for GSI specificity (Vieira et al., 2008, 2010; Aguiar et al., 2013, 2015). Under the non-self recognition by multiple factors model, the high intra-haplotypic diversity of *S*-pollen genes is the result of natural selection favoring diversification within an *S*-haplotype (Kakui et al., 2011; Aguiar et al., 2013, 2015; Pratas et al., 2018). Therefore, positively selected amino acid sites should be detected when carrying intra-haplotypic analyses (Aguiar et al., 2013, 2015; Pratas et al., 2018). Nevertheless, no strong evidence for positive selection is expected when individual *S*-pollen genes are considered (Vieira et al., 2009; De Franceschi et al., 2011a,b). A total of 16 amino acids under positive selection are here identified when performing codeML (Yang, 2007) analyses using 10 *Petunia S*-haplotypes having more than 11 genes characterized (Supplementary Figure S4; B+ database (bpositive.i3s.up.pt; see the *Petunia SLF* intra haplotype positive selection dataset BP2017000006). It should be noted that two of these amino acid sites are the same that Wu et al. (2018) assigned as being involved in specificity determination between *P. integrifolia* S2-SLF1 and S3-RNase. None of these amino acid positions are, however, those identified by Li et al. (2017), as determining the pollen *S* specificity *in vivo* between *S3-RNase* and *S3l-SLF1* in *P. hybrida*.

**FIGURE 2:**
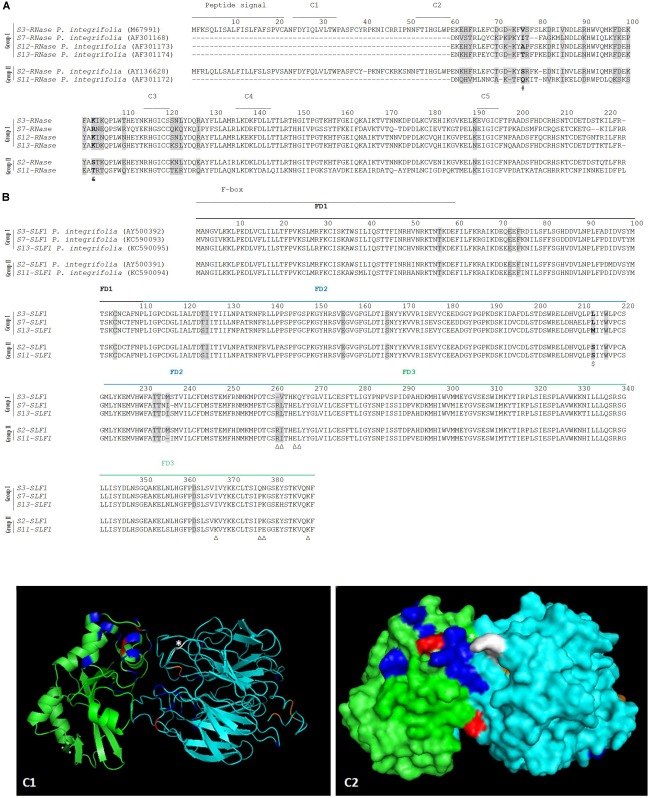
Positively selected amino acid sites in the S-RNase **(A)**, SLF1 **(B)** alignments (highlighted in gray), and in the predicted docking structure of *P. integrifolia* S3-RNase/S2-SLF1 (as cartoon C1, and surface C2) predicted to be involved in recognition of S3-, S7-, S12-, and S13-RNases (group I) as non-self, and S2-, and S11-RNases (group II) as self by the S2-SLF1 and S11-SLF1. **(A)** The signal peptide and conserved regions (C1- C5) are marked. The amino acid sites marked in bold are different between the two group of sequences regarding hydrophobicity (#), and charge and size (&). **(B)** The F-box and FD1-FD3 regions (Hua et al., 2007) are indicated. The amino acid site in bold and marked with a $ is different between the two groups of sequences in terms of hydrophobicity, size and polarity. Triangles indicate the eight putative amino acids at S2-SLF1 determining *P. integrifolia* S3-RNase specificity, according to Wu et al. (2018). **(C1,C2)** S3-RNase and S2-SFL1 are shown in green and in cyan, respectively. The amino acids under positive selection are highlighted in blue and red (in **A**- # and &; in **B**-$). The amino acids identified by Wu et al. (2018) are in orange and white if they are also here identified as positively selected. ^∗^Indicate amino acid sites under positive selection that are predicted to form hydrogen bonds and/or salt bridges between *P. integrifolia* S3-RNase and S2-SFL1.

**FIGURE 3:**
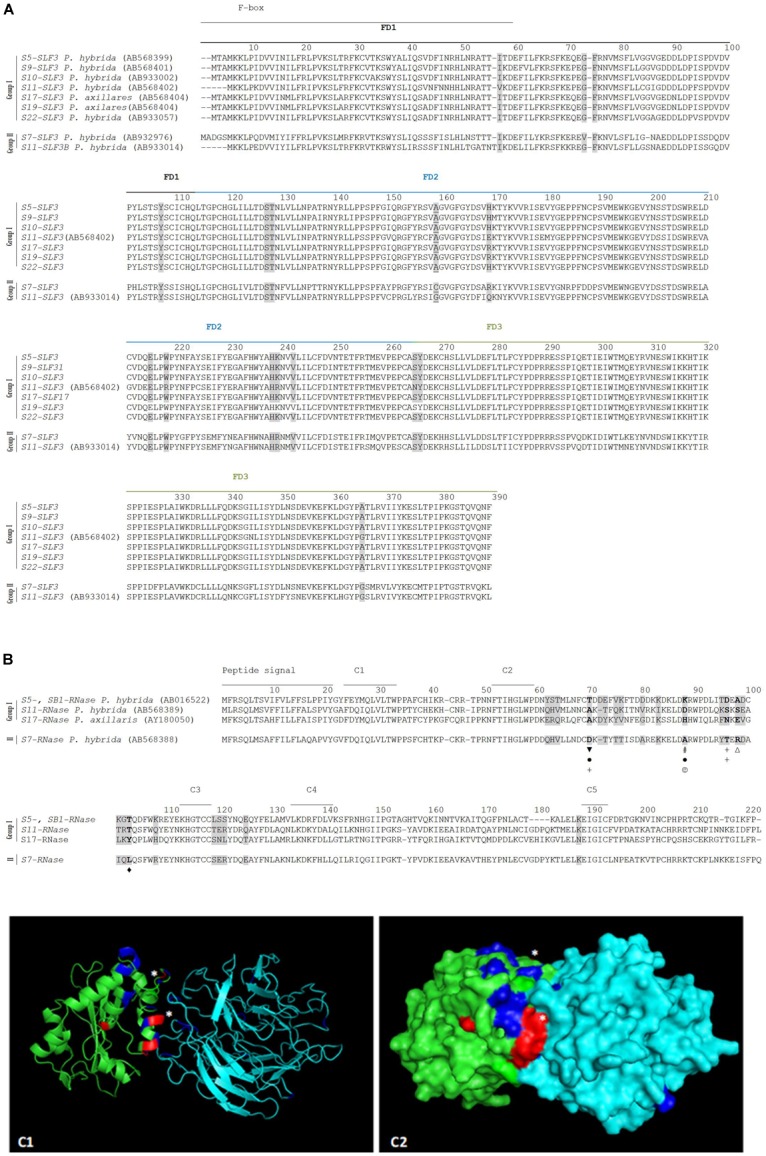
Positively selected amino acid sites at the SLF3 **(A)**, S-RNase **(B)** alignments (highlighted in gray), and in the predicted docking structure of *P. hybrida* S7-RNase/S5-SLF3 (as cartoon C1, and surface C2) predicted to be involved in the recognition of *S7-RNase* as non-self by the S5-, S9-, S10-, S11-, S17-, S19-, and S22-*SLF3* (that are highly conserved; Group I), and as self by S11-SLF3B and S7-SLF3 (group II). **(A)** The F-box and FD1-FD3 regions (Hua et al., 2007) are indicated. The amino acid site underlined is the only amino acid under positive selection different between the two groups of sequences. **(B)** The S7-RNase is different from S5-, S11-, and S17- RNases (group I) at five (marked in bold) amino acid sites under positive selection in terms of charge (marked with a 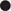, negatively charged 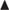, positively charged Δ), hydrophobicity (marked with a +), polarity (marked with a #), size (marked with a l’), and being aliphatic (marked with a 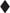). **(C1,C2)** *P. hybrida* S7-RNase/S5-SLF3 are shown in green and in cyan, respectively. The amino acids under positive selection are highlighted in blue and in red (those that show differences in hydrophobicity, or are aliphatic). ^∗^Indicate amino acid sites under positive selection that are predicted to form hydrogen bonds and/or salt bridges between *P. hybrida* S7-RNase/S5-SLF3.

Specificity determination implies physical interactions between the *S*-pollen and *S*-pistil proteins at particular amino acid sites. These amino acid sites may present proprieties such as hydrophobicity, size, charge, at a particular *S*-allele (or group of *S*-alleles) that allows to distinguish it (them) as non-self. Li et al. (2017) have shown that by swapping one amino acid between two SLFs, located in the C-terminal region (a region that contains a major specificity domain *in vivo*) that differs in charge and hydrophobicity, is sufficient to modify the specificity of the transgenic *P. hybrid*a S3-SLF1. Therefore, we looked for such amino acid properties at the sites identified as positively selected, by grouping sequences according to specificity recognition described in the literature. According to S2-SLF1 recognition we group the S3-, S7-, S12-, and S13-RNases (group I) as non-self S-RNases, and S2- and S11-RNases (group II) as self (Sun and Kao, 2013; Williams et al., 2014). The two groups of S-RNases differ in two amino acids identified as positively selected (marked in bold and gray in Figure 2A; (Vieira et al., 2007)), at position 76, and 103 (marked with a # and a & in Figure 2A) in what concerns hydrophobicity (group I is hydrophobic), and in the latter position, charge (group I is positively charged), and size (amino acids are small in group II). Dividing the *SLF1* sequences into the same groups, there are only two amino acid sites (213, and 262; Figure 2B) identified as positively selected that are different between the groups. The last position is one of the amino acids identified as determining *P. integrifolia* S2-SLF1/S3-RNase specificity, in Wu et al. (2018). It should be noted that, none of the other amino acid positions identified as putatively involved in specificity determination of *P. integrifolia* S2-SLF1/S3-RNase (marked with triangles in Figure 2B) are fixed in the groups here used. The amino acid located at position 213 is different in terms of hydrophobicity, size and polarity between the two groups (in group I is hydrophobic, and in group II is tiny and polar; marked with a $ in Figure 2B). In the predicted docking structure of *P. integrifolia* S2-SLF1/S3-RNase, these amino acids are located in the region of interface between the two proteins (those in red and white; Figure 2C1,C2). Therefore, these amino acid sites could be involved in the recognition of the S3-, S7-, S12-, and S13-RNases as self, and S2- and S11-RNases as non-self by S2- and S11-SLF1. It should be noted that, as expected, in the interface region of the two proteins are also located three of the amino acids identified by Wu et al. (2018), as putatively involved in *P. integrifolia* S2-SLF1/S3-RNase specificity (marked in orange and white those showing evidence for positive selection). According to Kubo et al. (2015), *S5*-, *S9*-, *S10*-, *S11*-, *S17*-, *S19*-, and *S22*-*SLF3* alleles, that are highly conserved, recognize as non-self the S7-RNase (although only the interaction between S7-RNase and S5- and S11-SLF3 has been confirmed in transformation experiments; group I), and only *S11-SLF3B* and *S7-SLF3*, two divergent *SLF3* alleles, recognize the S7-RNase as self (both cases are supported by transgenic experiments, group II; Kubo et al., 2015). According to the phylogenetic analyses here performed (Supplementary Figure S1) these two divergent *SLF3* sequences are a new gene (*SLF21*). When these groups of sequences are compared, amino acid 159 is the only site under positive selection that is different between the two groups (underlined in Figure 3A), and thus, could be involved in specificity determination of these sequences. When we compare the S5-, S11-, and S17- RNases (group I) and S7-RNase (group II) at the amino acid positions under positive selection, at amino acid positions 70, 88, 96, 98, and 103, there are differences in charge, hydrophobicity, polarity, size and being aliphatic (group I at amino acid position 70 is hydrophobic, at position 88 is polar and charged, at position 96 is hydrophilic, and in the group II at amino acid position 70 is negatively charged, at position 88 is tiny, at position 96 is hydrophobic, at position 98 is positively charged, and at position 104 is aliphatic; Figure 3B). These positions could be, in principle, involved in specificity recognition of the S7-RNase. When we look at these amino acids in the predicted docking structure of *P. hybrida* S7-RNase/S5-SLF3 (in red those that are positively selected and show different amino acid proprieties between the two groups; Figure 3C1,C2), these are located in the region of the interface between the two proteins. Therefore, these amino acid sites must be involved in the recognition of the *P. hybrida* S7-RNase as non-self by S5-SLF3.

**FIGURE 4:**
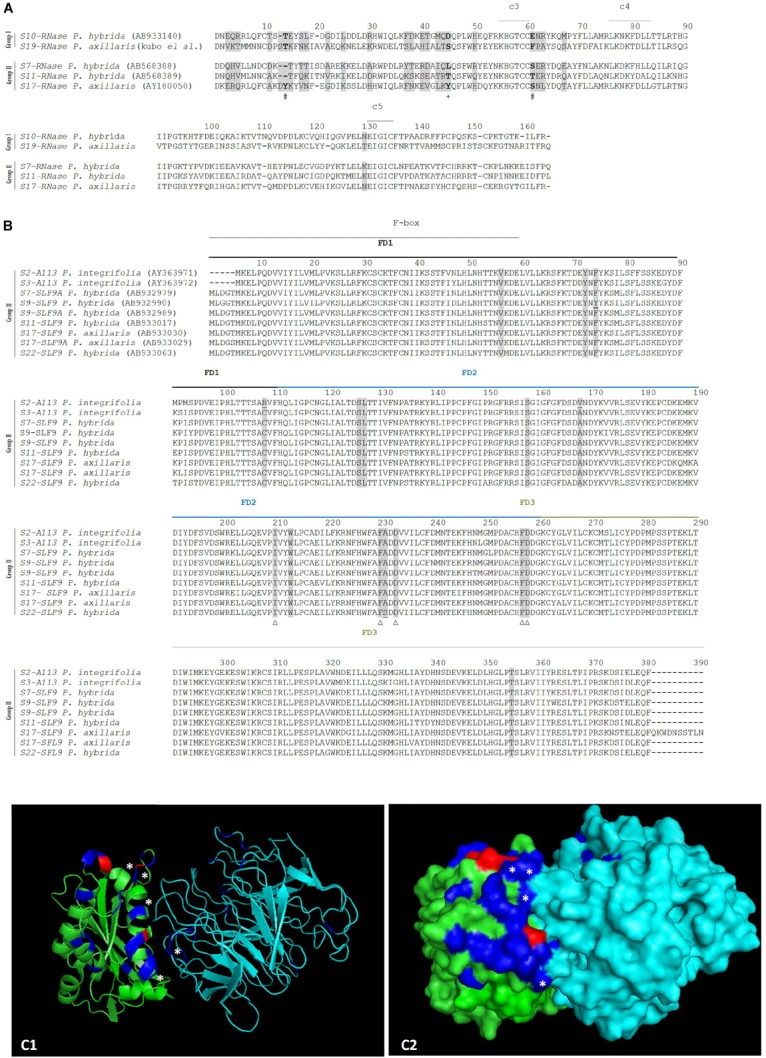
Positively selected amino acid sites at the S-RNase **(A)**, SLF9 **(B)** alignments (highlighted in gray), and in the predicted docking structure of *P. hybrida* S11-SLF9/S19-RNase (as cartoon C1, and surface C2) predicted to be involved in the recognition of *S10*-, and *S19-RNase* (group I) as non-self by the S2-, S3-S7-, S9-, S11-, S17-, and S22-SLF9A and SLF9B (group II), all showing high sequence conservation. **(A)** The conserved regions (C3- C5) are marked. The three amino acid positions under positive selection that are different between the two groups are highlighted in bold. Differences in size are marked with a #, and differences in hydrophobicity are marked with a +. **(B)** The F-box and FD1-FD3 regions (Hua et al., 2007) are indicated. The 12 amino acids under positive selection conserved in *SLF9A* and *SLF9B* alleles (those amino acids, highlighted in gray, that are not underlined) could be involved in specificity determination of S10- and S19-RNases. Those located in the region of the interface between the two proteins are marked with a Δ. **(C1,C2)** *P. hybrida* S19-RNase/S11-SLF9 are shown in green and in cyan, respectively. The amino acids under positive selection are highlighted in blue and in red (those that show differences in size and hydrophobicity). ^∗^Indicate amino acid sites under positive selection that are predicted to form hydrogen bonds and/or salt bridges between *P. hybrida* S19-RNase/S11-SLF9.

*S10*- and *S19-SLF9* alleles are missing, and according to Kubo et al. (2015), all type 9 sequences, that show high sequence conservation, can recognize both S10- and S19-RNases, although functional evidence is only available for S19-RNase and S7-, S11-SLF9. Type 9 sequences are, however, a heterogeneous group and represent four genes (Supplementary Figure S1). We consider all sequences from *SLF9A* and *SLF9B* genes as being able to recognize S10- and S19-RNases. When we group the available S-RNases accordingly (S10-, S19- RNases (group I) versus S7-, S11-, and S17-RNases (group II) there are three amino acid positions (14, 45, and 61; Figure 4A) that are different in size (positions 14 and 61) and hydrophobicity (position 45). When we group all available *SLF9* alleles there are 12 amino acid positions under positive selection that are conserved, and could be involved in specificity determination of S10- and S19-RNases (Figure 4B). Indeed, when we label them in the predicted docking structure of S11-SLF9/S19-RNase (Figure 4C1,C2), five (those marked with a star in Figure 4B), are located in the region of interface between the two proteins. The amino acid sites under positive selection that show differences in size and hydrophobicity at the S-RNase are also located in this region (Figure 4C1,C2). Therefore, these amino acid sites could be involved in the recognition of the S10-, S19-RNase as non-self by S2-, S3-, S7, S9, S11-, S17-, and S22-SLF9.

## Discussion

The number of *SLF* sequences identified in the different species seems to be more related with difficulties in assembling the genome at the *S*-locus region (due to the presence of highly repetitive DNA, such as transposable elements, for instance) than with the breeding system of the species analyzed. For instance, for the two SI *Petunia* species analyzed, although the coverage is as high as 91.3 and 90.2% of their diploid genomes^6^, only three *SLF* sequences have been identified. For extant species, SC is usually a recent event, since the average lineage duration of a SC species is on the order of 200 000 years (Goldberg et al., 2010), and thus, *SLF* sequences are expected to be retrieved in SC species, although many of them as pseudogenes. When this data is analyzed at the genus level, similar number of *SLF* gene lineages are described in *Solanum* (17), *Nicotiana* (19), and *Petunia* (18), although only eight are present in the three genera. It is possible that these *SLF* gene lineages have been devoted to the recognition of the same *S-RNases* in the three genera. The first studies on the characterization of *S-RNase* alleles from different Solanaceae species, showed evidence of broadly shared ancestral polymorphism (Ioerger et al., 1990; Richman and Kohn, 2000; Igic et al., 2004, 2006; Savage and Miller, 2006). Indeed, at least 83% of the *SLF* gene lineages were present in the Solanaceae common ancestor. Two (in *Nicotiana*) to three (in *Petrunia* and *Solanum*) *SLF* lineage genes seem to have appeared after the split of the genera. Thus, assuming one generation per year and the divergence time of 24 MY for the *Solanum* and *Nicotiana* genera, and 30 MY for the Solanaceae crown lineages (Sarkinen et al., 2013), the rate of origin of a new *SLF* gene lineage (and thus a new specificity) is one per 10 MY. This value fits the range for the rate estimates for the origination of new *S*-alleles [10^−6^ to 10^−9^ per gene per generation; (Vekemans and Slatkin, 1994)]. The presence of identical number of *SLF* gene lineages in the three genera suggests that in the non-self recognition system, 18 *SLF* genes is the minimum number of *S*-pollen genes for the system to work when about 30 different specificities (Tsukamoto et al., 2003) are present. This number is compatible with the simulation work performed by Kubo et al. (2015), that found that an average of 16–20 *SLFs* are enough to recognize the large majority of the *S-RNases* in *Petunia* populations. Since the number of *S*-haplotypes that can be maintained in a population depends of the effective population size, this also suggests no major differences in the effective population size of the species of the three genera. In Solanaceae, a population bottleneck has been suggested only for the common ancestor of *Physalis* and *Witheringia* (Paape et al., 2008) and in the genus *Lycium* (Miller et al., 2008).

Under the non-self recognition model an SLF interacts with a subset of S-RNases, but not with the self-S-RNase (Kubo et al., 2010, 2015; Williams et al., 2014, 2015). It has been proposed that recognition avoidance of the self-S-RNase is achieved by having either a diverged or deleted allele of the SLF type whose product recognizes the S-RNase of that S-haplotype (Kubo et al., 2015). The phylogenetic analyses here performed give support to this hypothesis.

*In vivo* transgenic assays revealed that different regions of the same allelic form of a SLF protein (S2-SLF1) are involved in specificity determination of the different S-RNases (Sijacic et al., 2004; Kubo et al., 2010; Sun and Kao, 2013; Williams et al., 2014; Li et al., 2017; Wu et al., 2018). For instance, FD3 region of *P. inflata* S2-SLF1 protein is required for the interaction with the *P. inflata* S3-RNase, and a maximum of four amino acid sites are involved in specificity determination, but FD2 region contains the amino acids that determine the specificity for *P. inflata* S7-RNase, and both FD1 and FD2 contain the amino acids that determine the specificity for *P. inflata* S13-RNase (Wu et al., 2018). Moreover, different allelic pairs of *P. hybrida* SLF1 (S3-SLF1 and S3L-SLF1 that interacts with *P. hybrida* S3-RNase), identify the FD3 region as containing the amino acids required for the interaction between S3L-SLF1 and S3-RNase (Li et al., 2017). Furthermore, one amino acid at position 293, when replaced with another with opposite electrostatic potential determines the interaction specificity of *P. hybrida* S3L-SLF1 and S3-RNase. Interactions between SLF proteins and S-RNases are, thus complex and diverse (Li et al., 2017). Such complexity implies that other methodologies need to be applied to predict those amino acids involved in specificity determination. Amino acid sites determining specificity are expected to be under a different selection regime than the rest of the protein, with selection favoring diversification within a *S*-haplotype (Kakui et al., 2011; Aguiar et al., 2013, 2015; Pratas et al., 2018). We identified 16 such amino acid positions, and two of these have been identified as involved in specificity determination between S2-SLF1 and S3-RNase in *P. integrifolia* (Wu et al., 2018). Nevertheless, our analysis was performed using only 10 *S*-haplotypes for which 11 *SLF* genes are available, and thus, not all amino acid sites under positive selection have been identified. Therefore, it is not surprising that the amino acid position identified as determining specificity of *P. hybrida* S3L-SLF1 and S3-RNase, has not been here identified (Li et al., 2017). These putative amino acid sites involved in specificity determination are located at the predicted interaction surface of the two proteins, and those at the S-RNase show distinct amino acid characteristics such as hydrophobicity, size and charge. Therefore, these amino acid positions show all characteristics of being involved in specificity determination. Using this methodology Pratas et al. (2018) identified the putative amino acid sites determining specificity in *Malus*. Therefore, this methodology can be applied to other species presenting GSI of the non-self recognition type. Although *S-RNase* and F-box genes belonging to the GSI lineage have been identified in Fabaceae (Aguiar et al., 2015; Ramanauskas and Igic, 2017), Rutaceae, and Malvaceae (Ramanauskas and Igic, 2017) species, further characterization of the *S*-locus, concerning levels of diversity, expression, segregation analyses and identification of positively selected amino acid sites, is needed to determine if GSI in such species is of the non-self recognition type, before applying the methodology here described.

## Data Availability

All datasets generated for this study are included in the manuscript and/or the Supplementary Files.

## Author Contributions

JV and CV collected the genome sequence data and performed the analyses. SR performed the analyses on the protein structures. MR-J, FF-R, NV, and HL-F designed and implemented the mentioned operations at BDBM. All authors wrote, read, and approved the final manuscript.

## Conflict of Interest Statement

The authors declare that the research was conducted in the absence of any commercial or financial relationships that could be construed as a potential conflict of interest.

## Footnotes

1 https://www.sing-group.org/BDBM/

2 http://bpositive.i3s.up.pt

3 https://www.ncbi.nlm.nih.gov/

4 https://www.sing-group.org/seda/

5 https://solgenomics.net/

6 https://www.nature.com/articles/nplants201674
