# Supplementary figures and images for: Predicting Specificities Under the Non-self Gametophytic Self-Incompatibility Recognition Model
Source: Front Plant Sci. 2019 Jul 4;10:879. doi: 10.3389/fpls.2019.00879 (PMC6649718; doi:10.3389/fpls.2019.00879)

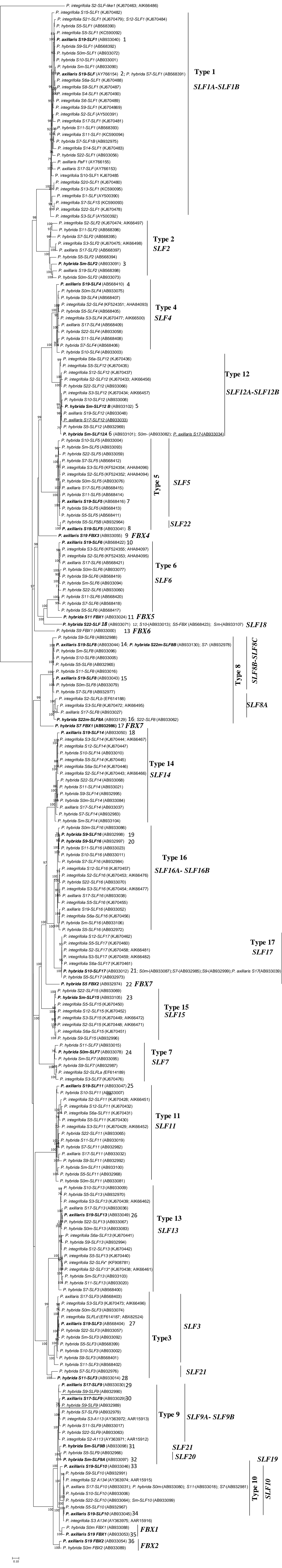

Supplement: Supplementary file 1 [file Image_1.pdf]

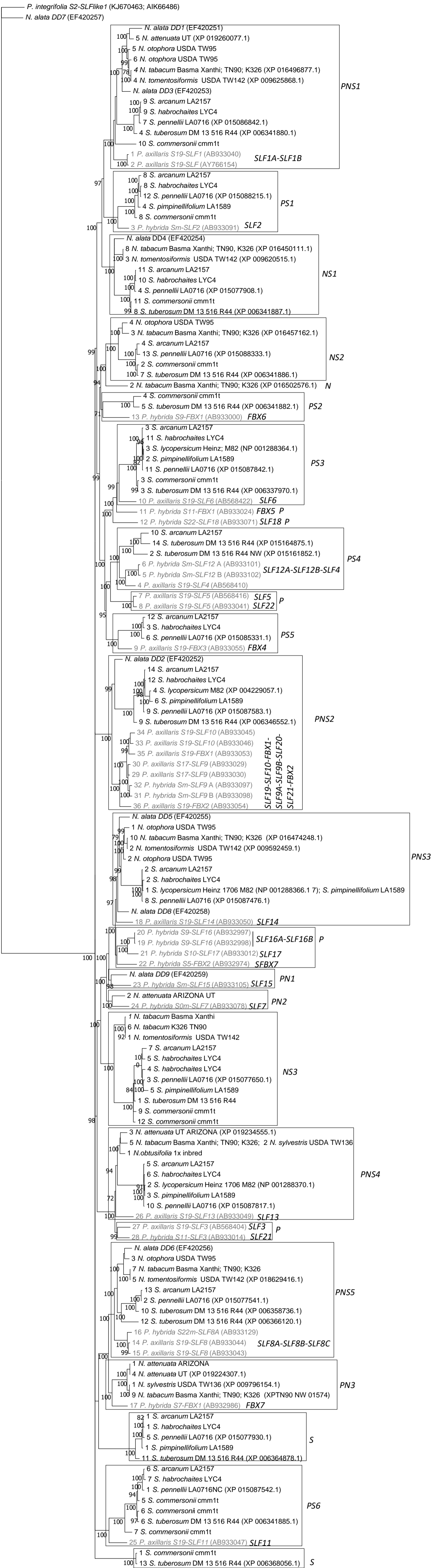

Supplementary Fig. S2

Supplement: Supplementary file 2 [file Image_2.pdf]

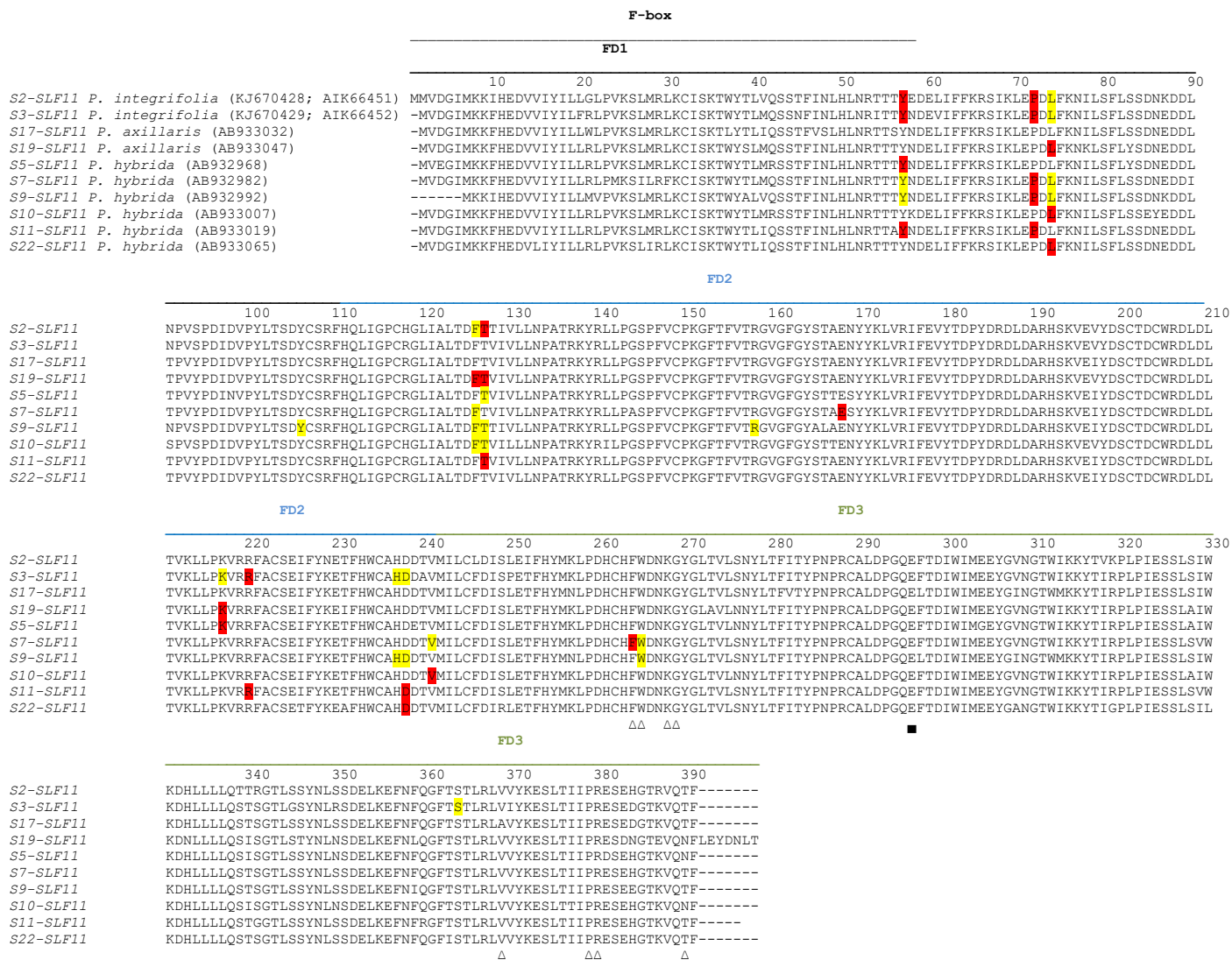

Supplemental Fig. S4

Supplement: Supplementary file 4 [file Image_4.pdf]
