# Supplementary material for: Predicting Specificities Under the Non-self Gametophytic Self-Incompatibility Recognition Model
Source: Front Plant Sci. 2019 Jul 4;10:879. doi: 10.3389/fpls.2019.00879 (PMC6649718; doi:10.3389/fpls.2019.00879)
Supplement: Supplementary file 3 [file Image_3.pdf]

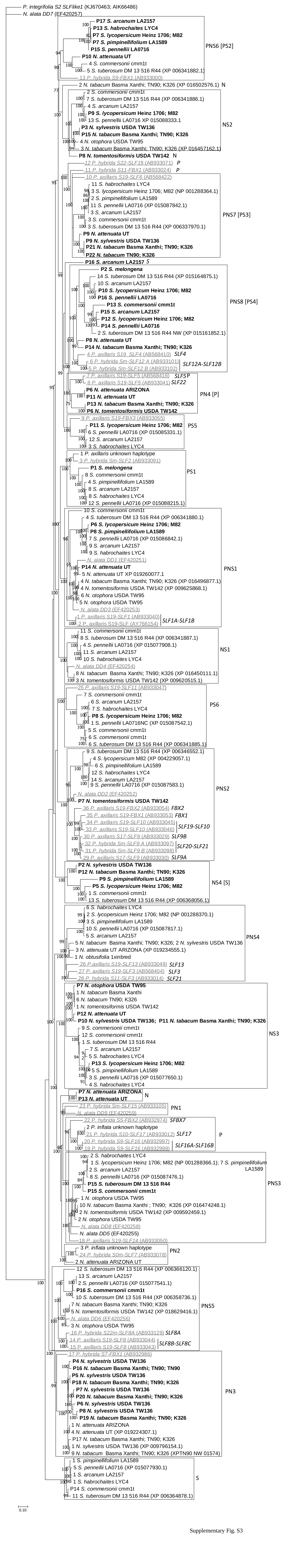

Supplementary Fig. S3
