# Supplementary material for: Predicting Specificities Under the Non-self Gametophytic Self-Incompatibility Recognition Model
Source: Front Plant Sci. 2019 Jul 4;10:879. doi: 10.3389/fpls.2019.00879 (PMC6649718; doi:10.3389/fpls.2019.00879)
Supplement: Supplementary file 5 [file Data_Sheet_1.PDF]

Supplementary Data for:

Predicting specificities under the non-self gametophytic self-incompatibility recognition model, by Jorge Vieira, Sara Rocha, Noé Vázquez, Hugo López-Fernández, Florentino Fdez-Riverola, Miguel Reboiro-Jato, and Cristina P. Vieira

Supplementary Fig. S1. Bayesian phylogenetic tree showing the relationship of the *Petunia SLF* sequences. Reference sequences are numerically marked and are in bold. The presence of two sequences from the same *S*-haplotype in the same phylogenetic group implies a gene duplication. Underlined sequences are sequences from the same *S*-haplotype that support a gene duplication already annotated. Numbers in the branches represent posterior credibility values above 70.

Supplementary Fig. S2. Bayesian phylogenetic tree showing the relationship of the *Petunia* reference sequences (in gray), *Nicotiana*, and *Solanum* functional *SLF* sequences. Squares represent gene lineages, and P, N, and S are used to mark if the gene lineage is present in *Petunia*, *Nicotiana*, and *Solanum*, respectively. Numbers in the branches represent posterior credibility values above 70.

Supplementary Fig. S3. Bayesian phylogenetic tree showing the relationship of the 211 Solanaceae *SLF* sequences. Sequences in bold, and assigned with a P, are putative pseudogene. Legend as in Figure 1.

Supplementary Fig. S4. Amino acid sites identified as positively selected (marked in colors) in intra-haplotype analyses of the 10 *Petunia S*-haplotypes for which more than 11 *SLF* genes are available (see B+ database (bpositive.i3s.up.pt; see the *Petunia SLF* dataset BP2017000006, for analyses). Sites were identified using the method of Yang (Yang, 1997) implemented in ADOPS (Reboiro-Jato *et al.*, 2012) with a probability higher than 95% in both NEB (naive empirical Bayes) and BEB (Bayes empirical Bayes) marked in yellow, or with a probability higher than 95% in NEB and 90% in BEB marked in red. The sites identified in each *S*-haplotype are marked in the SLF11 amino acid sequence of that *S*-haplotype. The F-box and FD1-FD3 regions (Hua *et al.*, 2007) are indicated. Triangles indicate the eight putative amino acids involved in specificity determination of *P. integrifolia* S3-RNase/S2-SLF1, according to Wu *et al.* (2018). The square

indicates the amino acid site involved in specificity determination in *P. hybrida* S3-RNase/ S3l-SLF1, identified by Li *et al.* (2017).
